# Reasons, Form of Ingestion and Side Effects Associated with Consumption of *Amanita muscaria*

**DOI:** 10.3390/toxics11040383

**Published:** 2023-04-17

**Authors:** Michal Ordak, Aleksandra Galazka, Tadeusz Nasierowski, Elzbieta Muszynska, Magdalena Bujalska-Zadrozny

**Affiliations:** 1 Department of Pharmacotherapy and Pharmaceutical Care, Faculty of Pharmacy, Medical University of Warsaw, Banacha 1 Str., 02-097 Warsaw, Poland; s080868@student.wum.edu.pl (A.G.);; 2 Department of Psychiatry, Medical University of Warsaw, 00-665 Warsaw, Poland; tadeusz.nasierowski@wum.edu.pl; 3 Department of Medical Biology, Medical University of Bialystok, 15-222 Bialystok, Poland; elzbieta.muszynska@umb.edu.pl

**Keywords:** *Amanita muscaria*, clinical symptoms, toxicology

## Abstract

In recent months, there has been a new trend involving the consumption of *Amanita muscaria*. The aim of this article was to investigate the reasons for consumption, the form taken and the adverse symptoms that were indicated by those consuming *Amanita muscaria*. After analysing 5600 comments, 684 people were included in the study, who, in social media groups such as Facebook, stated the purpose of consuming the mushroom (n = 250), the form of mushroom they were taking (n = 198) or the adverse symptoms they experienced (n = 236). The gender of the subjects differentiated the parameters analysed. In the study group of women, the main purpose of consuming *Amanita muscaria* was to reduce pain, as well as to reduce skin problems, while in men it was mainly to relieve stress, reduce the severity of depressive symptoms and reduce insomnia (*p* < 0.001). With regard to the form of mushroom ingested, tincture was predominant in the women’s study group, while dried was predominant in the men (*p* < 0.001). In terms of side effects, women reported primarily headaches, while men reported nausea, vomiting, abdominal pain and drowsiness (*p* < 0.001). Advanced research on *Amanita muscaria* should be carried out to make the community aware of the toxicity of this fungus.

## 1. Introduction

Since the mid-2000s, there has been a rapid emergence of a large and diverse group of new psychoactive substances (NPS), commonly referred to as “boosters” [1]. The introduction of a series of amendments to the Acts to add further NPS to its annexes has unfortunately not reduced the incidence of NPS abuse. We refer, for example, to the decision of the Council of the European Union, which subjected 4 methylmethcathinone (mephedrone) to control measures on 2 December 2010 [2]. Another example is the UK’s Psychoactive Substances Act 2016, which aimed, among other things, to eliminate NPS on the market [3]. For example, according to data published in Lancet Psychiatry, between 2010 and 2018, more and more patients were hospitalised each year for the abuse of mephedrone with other psychoactive substances [4]. The introduction of more and more laws related to drug policy has unfortunately contributed to an increase in the search for legal and readily available NPS. An example of this is the manganese encephalopathy resulting from the ingestion of ephedrone produced from drugs containing pseudoephedrine. A narrative review published in 2022 indicated that, among 93% of published case reports of patients abusing ephedrone, manganese concentrations were higher with respect to the current standard of 219 nmol/L [5]. Another example is the increasing prevalence of energy drink consumption. In a meta-analysis published in 2021, the authors included 32 studies and 96,549 individuals. The most common adverse effects included insomnia, stress and depressive mood. For this reason, it seems advisable to raise regulatory standards for the sale of energy drinks [6]. In recent months, there has been a new trend of this type, namely the regular consumption of *Amanita muscaria*. On a daily basis, many groups of people, including celebrities, post various posts encouraging the consumption of *Amanita muscaria*, which may consequently contribute to a number of symptoms. They are due to a combination of compounds showing biological activity, i.e., muscarinic, ibotenic acid and muscimol [7]. The articles available in the literature related to intoxication due to *Amanita muscaria* ingestion mainly concern case reports. It is generally accepted that *Amanita muscaria* is toxic to both sexes. Considering that the fungi is an active NPS drug, adverse effects are definitely shown if anybody consumes it in their daily diet. [7,8]. Ingestion of *Amanita muscaria* contributes to temporary agitation and depression of the central nervous system. The onset of symptoms begins between 30 min and 2 h and includes symptoms such as confusion, dizziness, agitation and ataxia, among others. Less commonly, nausea, diarrhoea, vomiting, tachycardia, bradycardia and hypertension may occur. Other possible symptoms include hypothermia or hyperthermia and metabolic acidosis [7,9]. Stereotactic injection of 5 µg/µL ibotenic acid into the hippocampus of injured rats contributes to cholinergic impairment of transmission, learning and memory [10]. Another exemplary article shows fatal poisoning by *Amanita muscaria* in a dog that had previously developed acute vomiting, diarrhoea, tremors, convulsions and lethargy. Analysis of stomach contents and urine revealed the presence of ibotenic acid and muscimol [11]. The duration of clinical symptoms averages 5–24 h [12]. These symptoms may also result from the presence of other compounds. Examples include toxic elements such as mercury, which may play a role in the development of visual and auditory hallucinations [13]. According to exemplary data published in the Journal of Environmental Science and Health, Part B, the topsoil from the upland location on which *Anamita muscaria* grew contained higher concentrations of mercury compared to the lowland layer [14]. Data on specific reasons for consuming *Amanita muscaria* are not observed in the literature. The data to date have mainly concerned single case reports in which the authors described symptoms associated with the consumption of this fungus. However, despite the growing trend associated with the consumption of *Amanita muscaria*, there is a lack of data on why this mushroom is consumed. For this reason, the main objective of this article was to analyse the social media views of the people surveyed who consume *Amanita muscaria* for the reasons they do so. An additional objective was to analyse the reported adverse effects and the forms in which *Amanita muscaria* is consumed.

## 2. Materials and Methods

### 2.1. Studied Group and Procedure

The study was carried out by analysing statements made by independent individuals in special Facebook groups. We are talking about groups of people who are advocates of consuming *Amanita muscaria*, who share different information every day. Other types of social media were not taken into account, due to the fact that it is on Facebook that there are many groups with *Amanita muscaria* in their name, to which the sample group belongs. This made it possible to select variables of interest in order to try to answer the question of for what purpose and in what form followers consume *Amanita muscaria* and what adverse effects they report. The parameters analysed included the following: the reason and the form of consumption of *Amanita muscaria*, as well as associated side effects. An additional extracted variable was the gender of the followers consuming *Amanita muscaria*. Age was not possible to analyse for the reason described in the limitations of this manuscript. Each of the extracted parameters was only included in the analysis if the proponent of *Amanita muscaria* consumption accurately stated the purpose, the form of the mushroom consumed and the associated adverse effects that followed. Individuals who imprecisely indicated information on one of these three aspects were not included in the analysis. An example would be a person sharing information that they do not know for what purpose they consume *Amanita muscaria*, perhaps for depression or perhaps still for skin problems. Other variables were not extracted, as the aim of the study, due to anonymity, was not to analyse the profiles of the proponents of *Amanita muscaria* consumption, but the three aspects mentioned above, which were the most frequently mentioned and therefore possible to analyse next. Each of these three parameters was analysed independently, i.e., they were not the same person. In other words, each parameter was analysed separately, as the statements made by the independents covered different aspects. A separate group of people commented on the purpose of consuming *Amanita muscaria*, separately on the form of intake, and separately still on the side effects. This has to do with the fact that one does not observe uniform responses from which one can concurrently deduce in one person why he or she consumed *Amanita muscaria*, the form of ingestion and possible side effects. For each parameter, the total frequency of responses was analysed, as well as the gender.

### 2.2. Statistical Analysis

Statistical analysis was performed using the SPSS25 statistical package (IBM SPSS Statistics Armonk, NY: IBM Corp.). A chi-square test was used to check whether the groups of people being compared were equal, as well as whether there was a statistically significant relationship between the parameters analysed and the gender of the people being studied. The effect size for statistically significant relationships was measured using Cramer’s V coefficient. A *p* value of < 0.05 was assumed as statistically significant.

## 3. Results

After analysing 5600 statements from people who were proponents of consuming *Amanita muscaria*, a group of 684 people (277 women—40.5% and 407 Men—50.5%) declaring regular intake of this mushroom were included in the analysis. 250 specified the specific purpose of this, 196 specified the form taken and 236 stated that they had a specific adverse symptom associated with the consumption of *Amanita muscaria*.

### 3.1. Reasons for Consumption of Amanita muscaria

In the groups of proponents of *Amanita muscaria* consumption, the statements of 250 people (108 women—43.2% and 142 Men—56.8%) who indicated a specific reason for their regular consumption of *Amanita muscaria* were analysed. The table below (Table 1) shows the individual reasons that were indicated in the group of 250 people. The most common reasons for consuming *Amanita muscaria* included stress relief, reduction of insomnia, as well as a reduction in the severity of depressive symptoms.

**Figure 1:**
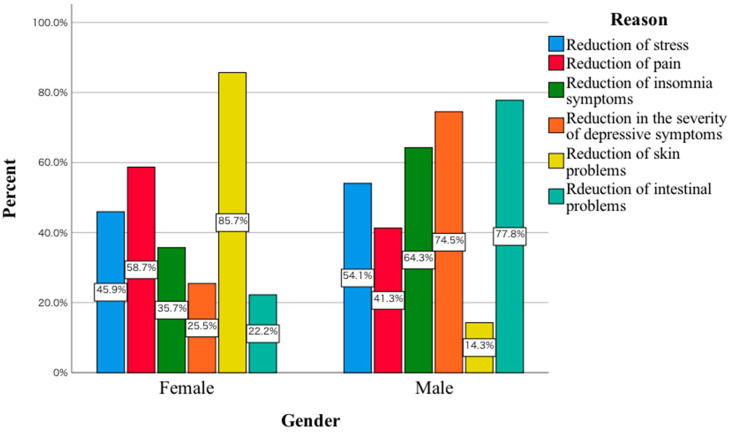
Reasons for consuming *Amanita muscaria* in the study group women and men.

A statistically significant relationship was observed between the gender of the subjects and the reason for their consumption of *Amanita muscaria*, χ^2^(5) = 24.45; *p* < 0.001; V_cr_ = 0.31. In the group of women surveyed, compared to men, a greater proportion said they consumed *Amanita muscaria* to reduce the severity of pain, as well as to reduce skin problems. In men, these were primarily for reasons such as stress relief, a reduction in the severity of depressive symptoms, as well as a reduction in insomnia (Figure 1).

### 3.2. Form of Consumption of Amanita muscaria

For the form of consumption of *Amanita muscaria*, the answers given by 198 people (92 women—39% and 144 Men—61%) were analysed. Three such forms have been identified, namely tincture, ambrosia and dried. A statistically significant higher proportion of the responses indicated consumption of *Amanita muscaria* in dried form, χ^2^(1) = 14.73; *p* < 0.001 (Table 2).

**Figure 2:**
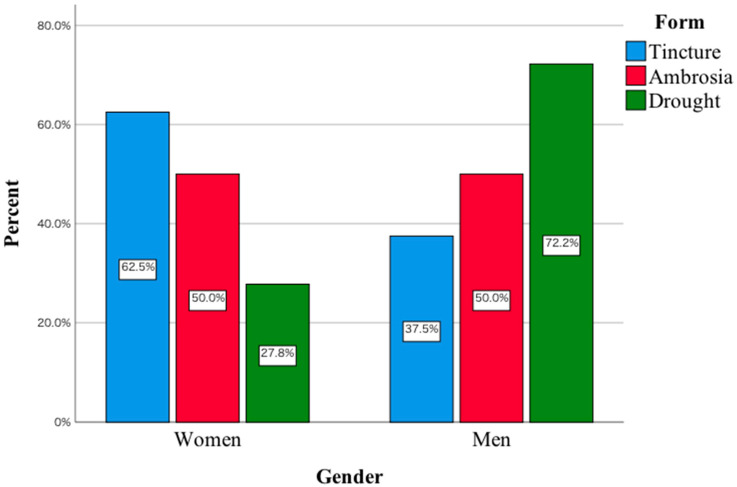
Form of consumption of *Amanita muscaria* in the study group women and men.

Gender also enters into a statistically significant relationship with the form of *Amanita muscaria* consumed, χ^2^(2) = 10.05; *p* < 0.001; V_cr_ = 0.31. In the women’s group studied, the main form was tincture, while in the men’s group, it was dried (Figure 2).

### 3.3. Adverse Effects Related to the Consumption of Amanita muscaria

The responses of 236 people (77 women—38.9% and 121 Men—61.1%) who indicated that they had experienced adverse effects related to the consumption of *Amanita muscaria* were also analysed. The most common such symptoms include lethargy, abdominal pain and nausea (Table 3).

**Figure 3:**
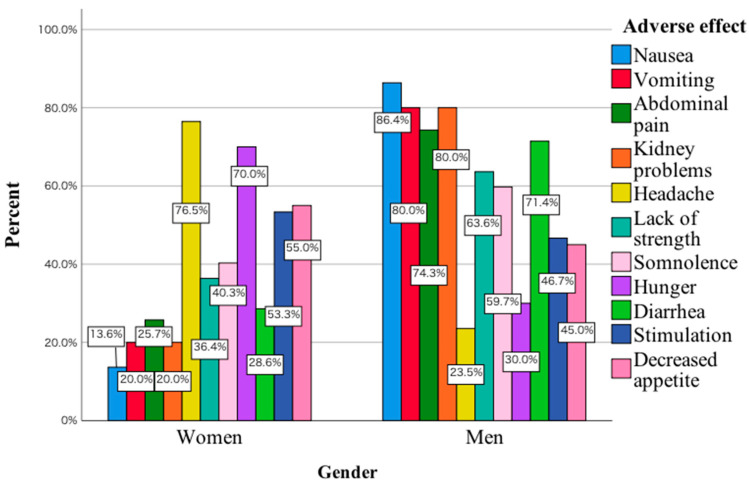
Adverse effects related to the consumption of *Amanita muscaria* in the study group women and men.

Adverse symptoms associated with *Amanita muscaria* consumption also enter into a statistically significant relationship with the gender of the subjects, χ^2^(10) = 29.83; *p* = 0.001; V_cr_ = 0.36. In the study group of women, the main adverse effects included headache, while in men, nausea, vomiting, abdominal pain and drowsiness were among the main adverse effects (Figure 3).

## 4. Discussion

According to data published in the International Journal of Environmental Research and Public Health, the pandemic has reduced the use of NPS in Poland. The majority of poisonings occurred in a group of men from large cities who use NPS socially and recreationally [15]. Changes in the law resulting in more difficult access to NPS have led to a search for more readily available substances. To the best of our knowledge, this is the first article to examine the recent trend associated with the consumption of *Amanita muscaria*, i.e., the reason, form and side effects associated with taking this mushroom. The articles published so far are mainly concerned with clinical symptoms in case reports of patients consuming *Amanita muscaria*. Observing every day the growing interest in this topic among various groups of people, including celebrities, we decided to analyse the aforementioned parameters by analysing 5600 comments, from which specific aspects were extracted.

The most common reasons for consuming *Amanita muscaria* in men included stress relief, reducing insomnia, as well as reducing the severity of depressive symptoms. In women, it was a reduction in the severity of pain, as well as a reduction in skin problems. Compared to women, more men are taking various types of new psychoactive substances, which has to do, among other things, with their greater willingness to take risks. Evidence can be found, for example, in results published in Lancet Psychiatry indicating that, among 601 patients abusing mephedrone, 93% were men [4]. One of the reasons indicating that men consume *Amanita muscaria* to alleviate psychiatric symptoms may be that they avoid admitting to emotional or psychiatric problems. They are less likely to report to specialists with symptoms of sadness, withdrawal from activities and lack of experiencing pleasure. Stigmatisation negatively affects men’s mental health help-seeking [16]. This may be the reason why men seek simpler ways to reduce depressive symptoms, including eating red toadstool. The men also indicated that they consume *Amanita muscaria* to reduce insomnia. This may have to do at least with the data published in the Journal of Gender Studies. The authors indicated that the use of strategies in coping with insomnia, as well as the perception of its severity, were higher in women compared to men [17]. In women, the consumption of *Amanita muscaria* to reduce skin problems is noteworthy. A study of 6630 women found that depressive symptoms and stress symptoms were significantly associated with skin disease [18]. In this study, women also indicated that they consumed *Amanita muscaria* to reduce headaches. In a 2021 review article, Pavlović pointed out that women are over-represented among patients presenting for headache treatment [19]. Women consuming *Amanita muscaria* probably have in mind that it will help to reduce the headaches they experience due to various life situations such as work-related stress, daily activities, etc. The form of *Amanita muscaria* consumed primarily was dried. In the surveyed group of women, the predominant form was tincture. As mentioned above, the argument explaining this fact may be that women are less likely to take risks compared to men. In a study of 954 behavioural trials, risk behaviour was measured repeatedly using three tests, namely refuge-use, thigmotaxis, and foraging latency. Men were found to be more adventurous in each of these tests compared to women [20]. The preparation of the tincture requires a more precise procedure, the use of which may reduce in women the anxiety associated with consuming another form of *Amanita muscaria*. The last parameter analysed was the adverse symptoms that were reported by the subjects after consuming red toadstool. The main symptoms include nausea, vomiting, abdominal pain and lethargy. In women, the main symptom was still headache. This is supported by the literature data to date indicating that gastrointestinal symptoms may occur during the first stage of *Amanita muscaria* ingestion, i.e., 6–12 h afterwards [21]. For example, an analysis of patients hospitalised at the Department of Toxicology in Poznan showed that the first symptoms included the occurrence of vomiting [22]. Articles published to date also indicate that drowsiness may occur after *Amanita muscaria* ingestion [11,22,23]. Drowsiness may be due to muscimol activity. In an article published in *Neurosurgery*, the authors indicated that higher concentrations of muscimol promoted the onset of somnolence [24]. It should also be noted that individual symptoms may be due to the presence of a number of other toxic elements. An example is the accumulation of speciation forms of vanadium, for which poisoning manifests itself, among other things, as irritation of the gastrointestinal tract [25,26]. Research to date has been based on investigating the toxic element content of *Amanita muscaria* in different parts of this fungus. There is a lack of research on the effect of different forms of *Amanita muscaria* preparation, including tinctures, on the concentration of these elements. In the article published in *Environmental Science and Pollution Research*, the authors point out in their conclusions that the loss of selenium and mercury during the processing of *Amanita muscaria* is not known [27]. For this reason, in the future, in order to thoroughly investigate the toxicity of *Amanita muscaria*, every form of consumed *Amanita muscaria* should be examined in detail for toxic elements, including ibotenic acid, muscarinic, muscimol and toxic elements. This would make it possible to determine which factors may actually be responsible for the clinical symptoms that occur in people who consume this fungus. It is also important to bear in mind the factor of seasonal variation, which can have a significant impact on the concentration of different elements. An example of this is a study published in *Biological Trace Element Research*, in which the authors compared mercury concentrations collected from the same medicinal plant sites in spring and autumn. In autumn, mercury concentrations were found to be statistically significantly higher compared to earlier in the year. In addition, the highest concentrations of this toxic element were found near busy streets [28]. For this reason, future research, in addition to taking into account seasonal variation, should also take into account where *Amanita muscaria* is harvested. Soil type is also another factor that needs to be taken into account, as, according to data published in 2021, this factor can influence toxic element concentrations [29]. It would then be possible to determine whether there is a relationship between the concentration of the analysed parameters in the soil and their concentration in *Amanita muscaria*. An argument pointing to the advisability of such a study is the fact that proponents of *Amanita muscaria* collect it in possibly different locations for this purpose. It would also be interesting to study the effects of *Amanita muscaria* intake on laboratory parameters, including those related to liver and kidney function. In other words, the effect of regular consumption of *Amanita muscaria* on the levels of key laboratory parameters related to organ function should be investigated in depth. For example, ibotenic acid and myscimol can induce acute renal failure [30]. At this point, studies conducted on a larger group of people are lacking. This includes a detailed study of the effects of *Amanita muscaria* consumed in various forms on levels of toxic elements. Some authors believe that the loss of ibotenic acid and muscimol properties occurs by cutting into small pieces and blanching *Amanita muscaria* [31]. However, there is no in-depth comparative analysis performed on the differences between the three forms of ingested *Amanita muscaria* mentioned in this manuscript with regard to e.g., toxic elements, ibotenic acid or e.g., still muscimol. The performance of interdisciplinary biological-chemical studies on *Amanita muscaria* and the soil on which this mushroom grows would allow the preliminary identification of toxic elements that may be responsible for, among other things, the adverse effects associated with the consumption of this mushroom. In addition to carrying out detailed chemical tests, including taking into account the seasonal variability of the harvested mushroom, it would be advisable in future to conduct media campaigns in which experts would convincingly discuss the toxicity of *Amanita muscaria*. This could contribute to reducing at least the sheer curiosity of those wishing to consume *Amanita muscaria*. According to recommendations published in European Psychiatry, in the case of mephedrone abuse, such campaigns may be one of the effective methods contributing to a reduction in the frequency of hospitalisations of patients taking this type of substance with alcohol and other drugs [32].

## 5. Conclusions

The focus these days should be on carrying out detailed research related to *Amanita muscaria*. This applies both to the individual parts of *Amanita muscaria* and to the forms of this mushroom consumed by the proponents. This will make it possible to identify which elements are responsible for the occurrence of clinical symptoms and to guard against possible consequences associated with their accumulation.

## 6. Limitations

A limitation of the present study is that it is not possible to obtain responses from one person on all the aspects analysed at the same time. The free statements made by people in Facebook groups relate only to single parameters, i.e., purpose, form or adverse effects associated with the consumption of *Amanita muscaria*. In other words, analysing the statements showed that people consuming *Amanita muscaria* do not post all the information at once, but only single pieces of information. For this reason, three independent groups of people were identified. The information extracted in addition to the three parameters indicated included only gender. Any data identifying a specific individual was not included in the analysis. Another limitation of the survey is that it is not possible to extract information on the age of the people surveyed. This type of information is rarely included in users’ Facebook profiles. The aim of the study was to extract possible information from social media so as to maintain full anonymity. A limitation of the study is also that it is an opinion-based survey and the population may not be right or have a scientific perception to some extent. The method of data collection may have limitations, such as potential sampling errors of people reporting on these Facebook groups or the reliability of self-reported data. However, during data collection, we attempted to reduce the risk of such error as much as possible by selecting responses specifically provided by proponents of *Amanita muscaria* consumption for analysis. The main limitation of the study, however, is the lack of an experimental approach. Performing the studies indicated in the discussion in the future would allow a broader view of the toxic elements contained in *Amanita muscaria*. However, in order to point out the need for such studies, this manuscript has been written, which, among other things, points out the adverse effects reported by those who consume *Amanita muscaria*. Demonstrating this growing trend seems to be one of the first steps towards undertaking further research in the coming years. In other words, the purpose of presenting the reasons, the form of *Amanita muscaria* consumed and the associated adverse effects, was to direct science towards the research mentioned in the discussion, i.e., the need for, inter alia, experimental studies.

## Figures and Tables

**Table 1:** Reasons for consumption of *Amanita muscaria* in the study group.

Reasons for Consumption of *Amanita muscaria*	n	%
Reduction of Stress	74	29.6
Reduction of Pain	46	18.4
Reduction of Insomnia Symptoms	56	22.4
Reducing the Severity of Depressive Symptoms	51	20.4
Reduction of Skin Problems	14	5.6
Reduction of Intestinal Problems	9	3.6

**Table 2:** Form of consumption of *Amanita muscaria* in the study group.

Form of Consumption of *Amanita muscaria*	n	%
Tincture	48	24.2
Ambrosia	24	12.1
Dried	126	63.6

**Table 3:** Adverse effects that occurred in the study group after consumption of *Amanita muscaria*.

Adverse Effects Related to the Consumption of *Amanita muscaria*	n	%
Nausea	22	9.3
Vomiting	15	6.4
Abdominal Pain	35	14.8
Kidney Problems	5	2.1
Headache	17	7.2
Lack of Strength	11	4.7
Somnolence	72	30.5
Hunger	10	4.2
Diarrhoea	14	5.9
Stimulation	15	6.4
Decreased Appetite	20	8.5

## Data Availability

The data presented in this study are available on request from the corresponding author.
